# Bayesian designs of phase II oncology trials to select maximum effective dose assuming monotonic dose-response relationship

**DOI:** 10.1186/1471-2288-14-95

**Published:** 2014-07-29

**Authors:** Beibei Guo, Yisheng Li

**Affiliations:** 1 Department of Experimental Statistics, , Baton Rouge, 70803 LA, USA; 2 Department of Biostatistics, The University of Texas MD Anderson Cancer Center, 1400 Pressler St, Unit 1411, Houston, 77030 TX, USA

**Keywords:** Bayesian hypothesis test, Bayesian model averaging, Nonlocal alternative prior density, Plateau, Efficacy, Toxicity

## Abstract

**Background:**

For many molecularly targeted agents, the probability of response may be assumed to either increase or increase and then plateau in the tested dose range. Therefore, identifying the maximum effective dose, defined as the lowest dose that achieves a pre-specified target response and beyond which improvement in the response is unlikely, becomes increasingly important. Recently, a class of Bayesian designs for single-arm phase II clinical trials based on hypothesis tests and nonlocal alternative prior densities has been proposed and shown to outperform common Bayesian designs based on posterior credible intervals and common frequentist designs. We extend this and related approaches to the design of phase II oncology trials, with the goal of identifying the maximum effective dose among a small number of pre-specified doses.

**Methods:**

We propose two new Bayesian designs with continuous monitoring of response rates across doses to identify the maximum effective dose, assuming monotonicity of the response rate across doses. The first design is based on Bayesian hypothesis tests. To determine whether each dose level achieves a pre-specified target response rate and whether the response rates between doses are equal, multiple statistical hypotheses are defined using nonlocal alternative prior densities. The second design is based on Bayesian model averaging and also uses nonlocal alternative priors. We conduct simulation studies to evaluate the operating characteristics of the proposed designs, and compare them with three alternative designs.

**Results:**

In terms of the likelihood of drawing a correct conclusion using similar between-design average sample sizes, the performance of our proposed design based on Bayesian hypothesis tests and nonlocal alternative priors is more robust than that of the other designs. Specifically, the proposed Bayesian hypothesis test-based design has the largest probability of being the best design among all designs under comparison and the smallest probability of being an inadequate design, under sensible definitions of the best design and an inadequate design, respectively.

**Conclusions:**

The use of Bayesian hypothesis tests and nonlocal alternative priors under ordering constraints between dose groups results in a robust performance of the design, which is thus superior to other common designs.

## Background

The maximum effective dose (MaxED), defined as the lowest dose that is effective (say achieves a pre-specified therapeutic target) and that also has full therapeutic effect [1], is important for various anti-cancer agents. For example, for traditional cytotoxic agents, for which the dose-response relationship is commonly assumed to be monotonically increasing, it is often of interest to identify the dose that achieves a ‘nearly maximal’ therapeutic effect, such as the ED95, the smallest dose at which 95% of the maximal response is achieved [2,3]. With the advent of molecularly targeted agents (MTAs) in recent years, identification of the MaxED becomes increasingly important as the efficacy of the MTAs may either increase or increase and then plateau in the tested dose range [4-9]. Based on considerations of long-term toxicity, potential shortages of drugs, and high costs, identifying the MaxED for further study is critical.

In practice, however, resource constraints may make it infeasible to test a large number of doses to accurately identify the MaxED. Consequently, in phase II trials, physicians often choose only two or three doses as an initial step to identify a potential lower dose that is sufficiently active and equally effective as higher doses. One common choice out of the small number of doses is either the maximum tolerated dose (MTD) or the highest tried dose from the phase I trials if the MTD was not identified (which tends to occur more frequently for an MTA than for a cytotoxic agent). This strategy is to ensure that the maximal benefit of the agent will be investigated. The remaining dose(s) chosen may be one or two levels below the chosen maximum dose, or even lower, if the preliminary efficacy at these doses from phase I trials supports these selections. Our goal in this paper is to propose designs for phase II oncology trials to identify the MaxED across a small number of doses, say two or three doses. These trials represent a preliminary step under limited resources toward the ultimate goal of identifying the MaxED. At The University of Texas MD Anderson Cancer Center, these trials are commonly proposed by physicians.

Our proposed designs are motivated by a phase II trial of a lysyl oxidase homolog 2 (LOXL2) inhibitor, an MTA, in adult patients with primary, post polycythemia vera, or post essential thrombocythemia myelofibrosis. Two doses were selected for evaluation of their best overall response. The physicians assumed that a higher dose would not lead to a lower efficacy or toxicity rate, yet a higher dose may not necessarily lead to a higher response rate. No dose-limiting toxicities (DLTs) had been observed from the phase I trials. The physicians decided to select two doses close to the lower and upper ends of the tested dose range from the phase I trials for evaluation in this phase II trial, based on the drug activity-related phase I and preclinical data. If the lower dose was found to confer equal benefit to the patients in the phase II trial, it would be used for further testing. Therefore, the goal of this trial was to identify the MaxED, restricted to two dose levels.

Based on the above motivating trial, we consider designs with a binary efficacy endpoint, for example, tumor response defined by complete or partial remission, or for certain MTAs, a pharmacodynamic response assessed by the change in relevant biomarker measurements that are considered to confer clinical benefit to the patients.

A number of authors have proposed designs for clinical trials relevant to the identification of the MaxED. For example, some authors considered designs for simultaneously identifying the minimum effective dose, the lowest dose that achieves a target anti-tumor effect, and the MaxED [2,10]. Other authors focused on designs for finding the MaxED when assuming a range of therapeutically useful doses has been established [11-13]. These methods are not applicable to our setting, where we aim to identify the MaxED only, without having already identified the minimum effective dose. Furthermore, the parametric models assumed in some of these designs [10,12] are unnecessary in our setting as we evaluate only two or three dose levels. Kong et al. [14] proposed a one-stage and a two-stage design to select the MaxED using isotonic regression and evaluate the efficacy of the selected MaxED. The objective of that design is similar to our objective; however, we focus on a binary efficacy endpoint, whereas they assume a continuous efficacy outcome.

To identify the MaxED, one approach is to use a frequentist multiple hypothesis testing procedure, in the spirit of Strassburger et al. [13], to evaluate whether one or more doses are sufficiently active and in cases where multiple doses are active, whether lower doses are equally effective as higher doses. A limitation of such a frequentist hypothesis test-based approach is its inability to declare that the null hypothesis (e.g., two doses being equally effective) is true, which can be critical for identifying the MaxED. Johnson and Cook [15] proposed a Bayesian hypothesis test with nonlocal alternative prior densities for designing single-arm phase II trials with continuous monitoring of futility and/or superiority. The nonlocal alternative prior densities refer to the prior densities used to define the alternative hypotheses that assign no mass to parameter values that are consistent with the null hypotheses. In contrast, the local alternative prior densities assign positive probability to regions of the parameter space that are consistent with the null hypotheses [15,16]. The Bayesian hypothesis test approach not only allows for direct evaluation of the probability of the null hypothesis (and of course the alternative hypothesis as well), but the use of the nonlocal alternative prior densities also allows a fair weight of evidence to be accumulated towards both the null and alternative hypotheses, thus facilitating a fair evaluation of both hypotheses [16]. This nonlocal alternative prior density approach, when applied to the design of clinical trials, also has an important *conservative* property in the sense that a specification of strong priors seemingly in favor of the treatment can actually decrease the expected weight of evidence collected in favor of the treatment [17]. This suggests the ‘objectivity’ of the approach by preventing cheating through the use of strong prior distributions in favor of the treatment. With these properties, their design was shown to outperform existing common single-arm phase II trial designs, including the Thall and Simon [18] and Simon two-stage designs [15,19].

The above features of the Bayesian hypothesis test with nonlocal alternative prior distributions motivate us to extend the approach to the design of phase II trials for identifying the MaxED. Specifically, due to the need to evaluate both the efficacy of more than one dose and potential equality of the efficacy between doses, we propose to extend the nonlocal alternative priors of Johnson and Cook [15] to those for multiple composite hypotheses with multivariate ordered parameters (such as priors in Hypotheses *H*_2_ and *H*_3_ in Section “Bayesian hypothesis testing”).

We propose an additional design based on Bayesian model averaging where nonlocal prior densities are used in models in which response probabilities are strictly ordered. We propose a continuous monitoring rule for each design, the Bayesian hypothesis test-based design and the Bayesian model averaging-based design, by evaluating the posterior probabilities of the multiple hypotheses and the posterior credible intervals for the response probabilities, respectively. In both designs, we implement a separate continuous monitoring rule for toxicity. Based on the motivating trial of the LOXL2 inhibitor, we use simulations to compare the performance of our proposed designs with those of three alternative designs.

The remainder of this article is organized as follows. In Section “Methods”, we present the two designs for two dose groups: the design based on Bayesian hypothesis tests in Section “Bayesian hypothesis testing” and that based on Bayesian model averaging in Section “Bayesian model averaging” we provide concluding remarks.

## Methods

### Bayesian hypothesis testing

Local prior densities that are used to define alternative hypotheses in most Bayesian tests are positive at values of the parameters that are consistent with the null hypothesis. As discussed in Johnson and Rossell [16], local alternative priors result in tests that provide exponential accumulation of evidence in favor of true alternative hypotheses, but only sublinear accumulation of evidence in favor of the true null hypothesis. In clinical trial designs, this means that the local alternative hypotheses result in designs that cannot terminate early in favor of a true null hypothesis. The nonlocal prior densities [16], by contrast, assign no mass to parameter values that are consistent with the null hypotheses. For example, the inverse moment prior densities used in Johnson and Cook [15] provide exponential convergence in favor of both the true null and true alternative hypotheses.

In this section, we consider using Bayesian hypothesis tests with nonlocal priors to facilitate the identification of the MaxED. Let *θ*_1_ and *θ*_2_ be the response rates at the lower and higher dose levels, and *θ*_0_ and *θ*_⋆_ be a response rate not of interest and the target response rate in the study, respectively. Both *θ*_0_ and *θ*_⋆_ can be specified in consultation with the study investigators. For example, *θ*_0_ can be the response rate of a standard treatment. With a lack of information, general guidelines are provided in Simon [19] to select appropriate values of the response rate that is not of interest and of the target response rate. We propose to equally randomize patients between groups, as it has been argued that outcome-adaptive randomization provides minimal benefit compared to equal randomization in trials with two or three arms [20,21]. We consider the following four hypotheses:

$$\begin{array}{lll} & H_{0}:\theta_{1}=\theta_{0},\theta_{2}=\theta_{0},\; & \\ & H_{1}:\theta_{1}=\theta_{0},\theta_{2}∼\pi_{\text{rI}}(\theta_{2};\theta_{0},k=1,\nu =2,\tau_{1}),\; & \\ & H_{2}:\theta_{1}=\theta_{2}∼\pi_{\text{rI}}(\theta_{1};\theta_{0},k=1,\nu =2,\tau_{1}),\; & \\ & H_{3}:(\theta_{1},\theta_{2})∼\pi_{\text{rI}}(\theta_{1};\theta_{0},k=1,\nu =2,\tau_{1})\; & \\ & \;\pi_{\text{rI}}(\theta_{2};\theta_{1},k=1,\nu =2,\tau_{2}),\; & \end{array}$$

where *π*_*rI*_(*θ*;*θ*^′^,*k*,*ν*,*τ*) = *π*_*I*_(*θ*;*θ*^′^,*k*,*ν*,*τ*) *I *(*θ*^′^ < *θ* < 1)/ *P *(*θ*^′^ < *θ* < 1) is the inverse moment prior *π*_*I*_(*θ*;*θ*^′^,*k*,*ν*,*τ*) [15] restricted on the interval (*θ*^′^,1) with

$$\begin{array}{lll} \pi_{I}(\theta ;\theta^{\prime },k,\nu ,\tau ) & =\frac{k\tau^{\nu /2}}{\Gamma (\nu /2k)}{\left[{\left(\theta -\theta^{\prime }\right)}^{2}\right]}^{-\frac{\nu +1}{2}}\; & \\ & \;\times \text{exp}\left\{-{\left[\frac{{\left(\theta -\theta^{\prime }\right)}^{2}}{\tau }\right]}^{-k}\right\}.\; & \; \end{array}$$

By equation (6) in Johnson and Cook [15], *P *(*θ*^′ ^< *θ *< 1) = 1/2 exp[-*τ *(1-*θ*^′^)^-2^] when *k *= 1 and *ν *= 2.

Under *H*_0_, both response rates are *θ*_0_, so neither dose level is promising. Under *H*_1_, only the higher dose level is promising. The corresponding response rate is assigned a nonlocal prior distribution with null value *θ*_0_. Under *H*_2_, both dose levels are promising, and the two response rates are the same. A nonlocal prior distribution with null value *θ*_0_ is used for the common response rate. Under *H*_3_, both dose levels are promising but the two response rates are different. To separate *H*_3_ from *H*_2_, we specify a nonlocal prior for *θ*_1_ with null value *θ*_0_ and a nonlocal prior to the difference in response rate, *θ*_2_-*θ*_1_, with null value 0. For all hypotheses, default values of *k*=1 and *ν *= 2 are used. *τ*_1_ can be chosen to result in a prior mode at *θ*_⋆_ and *τ*_2_ can be chosen to result in a prior mode at some level of difference in the response rate that is of interest to the study (e.g., 0.1). This suggests that the selection of *τ*_1_ and *τ*_2_ implicitly defines what is meant by a substantively meaningful difference between a response rate of interest and one not of interest, and between two response rates of interest [16].

The likelihood is L(θ1,θ2)=n1x1θ1x1(1-θ1)n1-x1×n2x2θ2x2(1-θ2)n2-x2. Under *H*_0_, the marginal likelihood is the likelihood evaluated at (*θ*_0_,*θ*_0_), i.e., *L *(*θ*_0_,*θ*_0_). Under *H*_1_, *H*_2_ and *H*_3_, we use one-, one-, and two-dimensional Monte Carlo integration to evaluate the marginal likelihood, respectively.

We first combine *H*_2_ and *H*_3_ to test three hypotheses: *H*_0_, *H*_1_, and $H_{2}^{⋆}=H_{2}\cup H_{3}$. $H_{2}^{⋆}$ represents the hypothesis in which both dose levels are promising, regardless of whether the two response rates are equal or not. Each patient is randomly assigned to one of the two dose levels with probability 0.5. Denote the prior model probability of *H*_0_, *H*_1_, *H*_2_ and *H*_3_ to be *P *(*H*_0_), *P *(*H*_1_), *P *(*H*_2_) and *P*(*H*_3_), respectively. After data from the first *n*_0_ patients are observed, we calculate the three posterior model probabilities according to $p(H_{0}|x):p(H_{1}|x):p(H_{2}^{⋆}|x)=p(H_{0})\times p(x|H_{0}):p(H_{1})\times p(x|H_{1}):p(H_{2}^{⋆})\times p(x|H_{2}^{⋆})$, where *n*_0_ is the minimum number of patients treated in the trial. In the absence of prior information about the probability of each hypothesis, we may assign *P*(*H*_0_) = *P*(*H*_1_) = *P*(*H*_2_) = *P*(*H*_3_) = 1/4 (as assumed in the BHT-A design in Section “Results and discussion”). In this case, $p(H_{0}|x):p(H_{1}|x):p(H_{2}^{⋆}|x)=p(x|H_{0}):p(x|H_{1}):p(x|H_{2})+p(x|H_{3})$. If instead we assume *P*(*H*_0_) = *P *(*H*_1_) = 1/3 and *P*(*H*_2_) = *P *(*H*_3_) = 1/6 (as assumed in the BHT-B design in Section “Results and discussion”), we have $p(H_{0}|x):p(H_{1}|x):p(H_{2}^{⋆}|x)=p(x|H_{0}):p(x|H_{1}):1/2\left[p(x|H_{2})+p(x|H_{3})\right]$. The trial is terminated if any of *p *(*H*_0_ ∣ *x*), *p*(*H*_1_ ∣ *x*) and $p(H_{2}^{⋆}|x)$ is greater than some threshold *P*_*a*_ or if the maximum sample size (*N*) is reached. If $H_{2}^{⋆}$ is concluded, then we conclude *H*_2_ if *p *(*x*∣*H*_2_)/*p *(*x*∣*H*_3_) > *P*_*b*_ and conclude *H*_3_ otherwise, with some threshold *P*_*b*_. If none of *p*(*H*_0_ ∣ *x*), *p*(*H*_1_ ∣ *x*) and $p(H_{2}^{⋆}|x)$ is greater than *P*_*a*_, then the above procedure is repeated after the outcome of each subsequently treated patient is observed. The trial is declared inconclusive if no hypothesis is concluded by the end of the trial.

In our simulations, *P*_*a*_ and *P*_*b*_ are set to be 0.65 and 1.2, respectively, in order to strike a balance between attaining overall high percentages of drawing correct conclusions at the end of the trial and reducing the sample size required for drawing the conclusions. The maximum sample size *N* is often chosen based on practical considerations such as budget constraints. Within the budget limit, *N* may be selected based on the physician’s judgment on the trade-off between the overall improved percentages for drawing the correct conclusions, as evaluated through simulations, and the time and resources required to achieve such improvements. The minimum sample size *n*_0_ is similarly chosen to strike such a balance. We prefer an average of 10 patients or more per dose level in order for the calculation of the posterior probabilities of the hypotheses to be meaningful. Such a choice is also consistent with the minimum sample size used in a design proposed by Thall and Simon for single-arm phase IIb trials [22]. We use simulations to select these and other design parameters to be introduced in later sections. In particular, we choose *n*_0_=24 across two groups (i.e., approximately 12 patients per group) in our simulation study.

We also monitor toxicity continuously during the trial. We first assign independent prior distributions to the probabilities of toxicity at the two dose levels, denoted as $p_{1}^{t}$ and $p_{2}^{t}$, respectively, based on information from the phase I trials. Next, to borrow strength (i.e., the ordering constraint) across the two dose levels, we apply a Bayesian isotonic regression transformation approach [23]. Specifically, for each pair $\left(p_{1}^{t},p_{2}^{t}\right)$ drawn from the unconstrained posterior distributions (e.g., under independent beta priors), the isotonic regression $\left(p_{1}^{t,⋆},p_{2}^{t,⋆}\right)$ of $\left(p_{1}^{t},p_{2}^{t}\right)$ is an isotonic function that minimizes the weighted sum of squares

$$\overset{2}{\underset{i=1}{\sum }}\omega_{i}{\left(\;p_{i}^{t,⋆}-p_{i}^{t}\right)}^{2}$$

subject to the constraint $p_{1}^{t,⋆}\leq p_{2}^{t,⋆}$[24]. The weights *ω*_*i*_ are taken to be the unconstrained posterior precision of $p_{i}^{t}$. It can be easily shown that $p_{1}^{t,⋆}$ and $p_{2}^{t,⋆}$ are weighted averages of $p_{1}^{t}$ and $p_{2}^{t}$ with weights *ω*_1_ and *ω*_2_ when the order between $p_{1}^{t}$ and $p_{2}^{t}$ is violated [24]. When the order is not violated, it is clear that $p_{1}^{t,⋆}=p_{1}^{t}$ and $p_{2}^{t,⋆}=p_{2}^{t}$. When there are three or more dose levels, we apply the pool-adjacent-violators algorithm [24] to obtain the order-restricted posterior samples. Suppose that the toxicity upper limit is $\bar{p}$. Define dose level *i* to be toxic if $p\left(p_{i}^{t,⋆}>\bar{p}|\text{data}\right)>P_{c}$ for some threshold *P*_*c*_. After the toxicity outcome of each patient is observed, we terminate the trial if both dose levels are toxic and close the higher dose arm if only the higher dose is toxic.

### Bayesian model averaging

In the second design we propose, we also assume that both the response rates and probabilities of toxicity are ordered across the two dose levels. We similarly use equal randomization between groups. This design is based on the calculation of posterior credible intervals. Our goal is to determine whether the two dose levels are promising or not, and whether the two response rates are the same when both dose levels are found promising. We consider two models — *M*_1_: *θ*_1_ = *θ*_2_ and *M*_2_ : *θ*_1_ < *θ*_2_. Under *M*_1_, the prior distribution is *θ*_1_ = *θ*_2_∼Uniform(0,1). Under *M*_2_, we assume *θ*_1_ ∼ Uniform(0,1) and *θ*_2_ ∣ *θ*_1_ ∼ *π*_*rI *_(*θ*_2_;*θ*_1_,*k* = 1,*ν* = 2,*τ*_2_)*I*(*θ*_1_ < *θ*_2_ < 1) with *τ*_2_ chosen so that the prior mode is at some value of the difference between *θ*_2_ and *θ*_1_ that is of interest to the study, say 0.1. Thus, the joint prior distribution of *θ*_1_ and *θ*_2_ is proportional to

$${\left[{\left(\theta_{2}-\theta_{1}\right)}^{2}\right]}^{-\frac{\nu +1}{2}}\text{exp}\left\{\;-{\left[\frac{{\left(\theta_{2}-\theta_{1}\right)}^{2}}{\tau }\right]}^{-k}\right\}I(0<\theta_{1}<\theta_{2}<1).$$

Under *M*_1_, the marginal likelihood *p *(*x *∣ *M*_1_) has a closed form: n1x1n2x2B(x1+x2+1,n1+n2-x1-x2+1), where *B *(·,·) is the beta function. Under *M*_2_, the marginal likelihood *p *(*x* ∣ *M*_2_) does not have a closed form, so we use a two-dimensional Monte Carlo integration to evaluate the marginal likelihood. Note that in the above prior specification, the Uniform(0,1) distribution may be replaced by a vague beta distribution with a mean consistent with a physician’s prior guess of the response rate under each model. We assign equal prior probabilities to the two models: *p*(*M*_1_) = *p*(*M*_2_) = 1/2. Each patient is randomly assigned to one of the two dose levels with probability 0.5. After the data from a minimum of *n*_0_ patients are observed, we calculate the two posterior model probabilities according to the formula *p*(*M*_1_ ∣ *x*) : *p *(*M*_2_ ∣ *x*) = *p *(*x *∣ *M*_1_) : *p *(*x *∣ *M*_2_). Consider three posterior probabilities: *p*_1 _= *P *(*θ*_1 _> *θ*_⋆ _- *δ*, *θ*_2_ > *θ*_⋆ _- *δ *∣ data), *p*_2_ = *P *(*θ*_1_ < *θ*_0 _+ *δ*, *θ*_2 _> *θ*_⋆ _- *δ *∣ data), and *p*_3 _= *P*(*θ*_1 _< *θ*_0 _+ *δ*, *θ*_2 _< *θ*_0 _+ *δ *∣ data), where *δ* is a small positive value. The trial is terminated if the maximum sample size is reached, *p*_1_>*P*_*e*_, or *p*_2 _+ *p*_3 _> *P*_*f*_, where *p*_1_ is the probability averaged over the two models:

$$\begin{array}{ll} p_{1} & =p(\theta_{1}>\theta_{⋆}-\delta ,\;\theta_{2}>\theta_{⋆}-\delta |\text{data})\\ & =\overset{2}{\underset{k=1}{\sum }}p(\theta_{1}\;>\theta_{⋆}\;-\;\delta ,\;\theta_{2}\;>\theta_{⋆}\;-\;\delta \;|\;M_{k},\text{data})p(M_{k}\;|\;\text{data}), \end{array}$$

and similarly for the other two probabilities. If the trial is not terminated, this procedure continues after the outcome of each subsequently treated patient is observed. At the end of the trial, if *p*_1 _> *P*_*e*_, we conclude $H_{2}^{⋆}$; we claim *H*_2_ if *p *(*M*_1 _∣ data)/*p *(*M*_2 _∣ data) > *P*_*g*_, and *H*_3_ otherwise. If *p*_2 _+ *p*_3 _> *P*_*f*_, we conclude *H*_0_ if *p*_2 _- *p*_3 _≤ *P*_*k*_, and conclude *H*_1_ otherwise. *P*_*e*_ and *P*_*f*_ are similarly chosen based on simulations, as for *P*_*a*_, *P*_*b*_, etc.

As in the previous section, we use Bayesian isotonic regression transformation to compute and monitor toxicity continuously. If both dose levels are considered to be excessively toxic, the trial is terminated; if only the high dose level is toxic, then this dose group is closed.

### Three-dose designs

So far, we have described the designs for two dose groups. Our designs can be extended to multiple dose groups in a straightforward fashion. For example, suppose three doses are being evaluated. Let *θ*_1_, *θ*_2_ and *θ*_3_ be the response rates at the low, median, and high dose levels, respectively. In the Bayesian hypothesis testing (BHT) approach, we consider the following hypotheses:

$$\begin{array}{lll} & H_{0}:\theta_{1}=\theta_{2}=\theta_{3}=\theta_{0}\; & \\ & H_{1}:\theta_{1}=\theta_{2}=\theta_{0},\theta_{3}∼\pi_{\text{rI}}(\theta_{3};\theta_{0},k=1,\nu =2,\tau_{1})\; & \\ & H_{2}:\theta_{1}=\theta_{0},\theta_{2}=\theta_{3}∼\pi_{\text{rI}}(\theta_{2};\theta_{0},k=1,\nu =2,\tau_{1})\; & \\ & H_{3}:\theta_{1}=\theta_{0},(\theta_{2},\theta_{3})∼\pi_{\text{rI}}(\theta_{2};\theta_{0},k=1,\nu =2,\tau_{1})\; & \\ & \;\pi_{\text{rI}}(\theta_{3};\theta_{2},k=1,\nu =2,\tau_{2})\; & \;\\ & H_{4}:\theta_{1}=\theta_{2}=\theta_{3}∼\pi_{\text{rI}}(\theta ;\theta_{0},k=1,\nu =2,\tau_{1})\; & \\ & H_{5}:\theta_{1}∼\pi_{\text{rI}}(\theta_{1};\theta_{0},k=1,\nu =2,\tau_{1}),\theta_{2}=\theta_{3}\; & \\ & \;∼\pi_{\text{rI}}(\theta_{2};\theta_{1},k=1,\nu =2,\tau_{1})\; & \\ & H_{6}:\theta_{1}=\theta_{2}∼\pi_{\text{rI}}(\theta_{1};\theta_{0},k=1,\nu =2,\tau_{1}),\theta_{3}\; & \\ & \;∼\pi_{\text{rI}}(\theta_{3};\theta_{2},k=1,\nu =2,\tau_{1})\; & \\ & H_{7}:\theta_{1}∼\pi_{\text{rI}}(\theta_{1};\theta_{0},k=1,\nu =2,\tau_{1}),\; & \\ & \;\theta_{2}∼\pi_{\text{rI}}(\theta_{2};\theta_{1},k=1,\nu =2,\tau_{1}),\; & \\ & \;\theta_{3}∼\pi_{\text{rI}}(\theta_{3};\theta_{2},k=1,\nu =2,\tau_{1})\; & \end{array}$$

Upon assigning appropriate prior probabilities to the eight hypotheses, final conclusions and interim monitoring are based on the evaluation of the posterior probability of each hypothesis.

In the Bayesian model averaging (BMA) approach, there are four possible models:

$$\begin{array}{lll} & M_{1}:\theta_{1}=\theta_{2}=\theta_{3}\; & \\ & M_{2}:\theta_{1}=\theta_{2}<\theta_{3}\; & \\ & M_{3}:\theta_{1}<\theta_{2}=\theta_{3}\; & \\ & M_{4}:\theta_{1}<\theta_{2}<\theta_{3}\; & \end{array}$$

The computation is parallel to that of the two-dose case.

As the number of doses increases, the number of hypotheses/models increases rapidly. So our proposed methods should be most efficient when there is a small number of (such as two or three) doses being tested, as is often the case for practical phase II trials with a goal of identifying the MaxED when assuming ordered response rates.

## Results and discussion

### Design operating characteristics

We evaluate the performance of the proposed designs based on the motivating trial of the LOXL2 inhibitor. The study drug is hypothesized to exert a therapeutic effect in fibrosis and cancer by inhibiting fibroblast activation and thereby altering the pathologic matrix in different disease states. The consequences of inhibiting fibroblast activation include substantial reduction of desmoplasia, decreased expression of growth factors and cytokines, lack of formation of tumor vasculature, and increased necrosis, pyknosis, and autophagy of tumor cells. Given the hypothesized action of this MTA, the investigators assumed that efficacy either increases or increases and then plateaus in the tested dose range. Toxicity was assumed to be nondecreasing with an increasing dose. Two dose levels were to be evaluated, and a maximum of 54 patients were to be enrolled. The primary goal of the study was to determine whether both dose levels would result in a target response rate of greater than or equal to 30*%* against a null hypothesis of 10*%*. If both doses achieved the target response rate and appeared comparable, the investigators would proceed with the lower dose for subsequent testing. However, if activity was primarily seen at the higher dose level, or if both doses achieved the target response rate, yet the higher dose had a considerably higher response rate, the investigators would test the higher dose in subsequent studies.

In this study, response was defined as the clinical response based on the International World Group criteria. In particular, stable disease with improvement in bone marrow fibrosis score, clinical improvement, partial remission, or complete remission would be considered a response. Toxicity was defined as dose-limiting toxicity (DLT) with pre-specified categories and grades. In the corresponding phase I study, four dose levels had been evaluated in patients with advanced solid tumors, and three patients had been treated at each dose level. Given a patient’s weight of 70 kg, the two middle dose levels tested in the phase I trial were very close to the dose levels considered in this study. As no DLTs or drug-related severe adverse events had been observed at any dose in the phase I trial, to elicit informative prior distribution for toxicity at a given dose by incorporating toxicity data at the same and higher dose levels from the phase I trial, we chose to treat every three patients at the higher dose level without toxicity as five patients at the lower dose level without toxicity, when the lower doses were studied in this phase II trial. Our final prior distributions for the probabilities of toxicity were $p\left(p_{1}^{t}\right)∼\text{beta}(0.5,16.5)$ and $p\left(p_{2}^{t}\right)∼\text{beta}(0.5,8.5)$, both of which were obtained by assuming a beta(0.5,0.5) prior distribution prior to observing the phase I toxicity data.

We first compare our proposed designs with two alternative designs, both of which use futility and efficacy continuous monitoring rules. The first design is an independent design that uses Bayesian hypothesis tests with a nonlocal alternative prior [15] at each dose level. The null and alternative hypotheses at the two dose levels are

$$H_{0}^{i}:\theta_{i}=\theta_{0},\;H_{1}^{i}:\theta_{i}∼\pi_{\text{rI}}(\theta_{i};\theta_{0},k=1,\nu =2,\tau_{1}),$$

for *i *= 1,2. Arm *i* (*i *= 1,2) is terminated for efficacy if $p(H_{1}^{i}|x)>P_{a⋆}$, and is terminated for futility if $p(H_{0}^{i}|x)>P_{a⋆}$, with *P*_*a *⋆ _> 0.5 being a cutoff value to be tuned by simulations. We conclude *H*_0_ if both $H_{0}^{1}$ and $H_{0}^{2}$ are found to hold; we conclude *H*_1_ if $H_{0}^{1}$ and $H_{1}^{2}$ are found to hold; and we conclude $H_{2}^{⋆}$ if $H_{1}^{1}$ and $H_{1}^{2}$ are found to hold. For these independent designs, we cannot obtain an exact posterior probability that the two response rates are equal, so we use approximations. If $H_{2}^{⋆}$ holds, then we conclude *H*_2_ if *p *(*θ*_2 _- *θ*_1 _> 0.1 ∣ *x*) ≤ *P*_*d*_, and conclude *H*_3_ otherwise, with *P*_*d*_ being some threshold to be calibrated by simulations. We assign independent prior distributions for the toxicity probabilities at the two dose levels, and terminate the trial if both dose levels are toxic and close either arm if the corresponding dose level is toxic.

The second design we assess for comparison is based on Bayesian isotonic regression transformation (BIT) [23]. The prior distributions are independent Uniform(0,1) distributions for both *θ*_1_ and *θ*_2_. After data are observed, the unconstrained posterior distributions of *θ*_1_ and *θ*_2_ are independent beta distributions. For each pair of (*θ*_1_,*θ*_2_) drawn from the unconstrained posterior beta distributions, the order-restricted posterior samples $\left(\theta_{1}^{⋆},\theta_{2}^{⋆}\right)$ are obtained as weighted averages of (*θ*_1_,*θ*_2_) when the order is violated, or otherwise remain unchanged, where the weights *ω* are proportional to the unconstrained posterior precision at the two dose levels. Consider three posterior probabilities: *p*_1 _= *P *(*θ*_1 _> *θ *_⋆ _- *δ*, *θ*_2 _> *θ*_⋆ _- *δ *∣ data), *p*_2 _= *P *(*θ*_1 _< *θ*_0 _+ *δ*, *θ*_2 _> *θ*_⋆ _- *δ *∣ data), and *p*_3 _= *P *(*θ*_1 _< *θ*_0 _+ *δ*, *θ*_2 _< *θ*_0 _+ *δ *∣ data). The trial is terminated if the maximum sample size is reached, *p*_1 _> *P*_*h*_, or *p*_2 _+ *p*_3 _> *P*_*i*_. This procedure is undertaken after the outcome of each subsequently treated patient is observed. At the end of the trial, if *p*_1 _> *P*_*h*_, we conclude $H_{2}^{⋆}$, and claim *H*_2_ if *p *(*θ*_1 _= *θ*_2 _∣ data) ≥ *p *(*θ*_1 _< *θ*_2 _∣ data), and *H*_3_ otherwise. If *p*_2 _+ *p*_3 _> *P*_*i*_, we conclude *H*_0_ if *p*_2 _- *p*_3 _≤ *P*_*j*_, and conclude *H*_1_ otherwise. The rule for early termination due to toxicity is the same as in the BHT approach.

Given that little toxicity was found in the phase I studies, we assumed low toxicity probabilities in our simulation scenarios, specifically, 0.15 at both dose levels. The upper limit of the toxicity probability $\bar{p}=0.3$. The null and target response rates are *θ*_0 _= 0.1 and *θ*_⋆ _= 0.3. We chose *τ*_1 _= 0.06 and *τ*_2 _= 0.015 to correspond with a prior mode at 0.3 and a value of interest, the between-dose difference in response rate of 0.1. The between-dose difference of interest refers to a minimal clinically meaningful difference between doses. To facilitate the comparison of the performances of several methods, the cutoffs of each method were chosen to approximately match the resulting type I error and average sample size under *H*_0_, based on simulations. The cutoffs used were *P*_*a *_= 0.65, *P*_*b *_= 1.2, *P*_*c *_= 0.8, *P*_*a *⋆ _= 0.7, *P*_*d *_= 0.11, *P*_*e *_= 0.7, *P*_*f *_= 0.65, *P*_*g *_= 1.3, *P*_*h *_= 0.65, *P*_*i *_= 0.65, *P*_*j *_= 0.4, *P*_*k *_= 0.02, and *δ *= 0.1. For the independent design, the maximum sample size was set at 27 at each dose level. For all designs, we used a minimum total sample size of 24 (12 at each dose level for the independent design) as “burn-in”, and continuous monitoring of futility and efficacy after the burn-in period. In addition, we monitored toxicity continuously starting from the first patient. We constructed 12 scenarios with different true response rates at the two dose levels. Under each scenario, we simulated 1,000 trials.

The operating characteristics of the four designs are summarized in Table 1, with the joint BHT design labeled ‘BHT-A’, the independent BHT design labeled ‘indep’, the BMA design ‘BMA’, and the BIT design ‘BIT’. We also conducted a sensitivity analysis to evaluate different prior probabilities of the four hypotheses under the joint BHT design. Instead of assuming equal prior probability of each of the four hypotheses, we gave equal prior probability for the three hypotheses, i.e., 1/3, to *H*_0_, *H*_1_, and $H_{2}^{⋆}$, and 1/6 probability to each of *H*_2_ and *H*_3_. The corresponding results are shown in Table 1 under column ‘BHT-B’. Under each scenario, we list the true response rates at the two dose levels in the top row, the probability of concluding that each of the four hypotheses is true in the next four rows, the probability of concluding that both dose levels are promising in the sixth row, and the average sample size and percentage of inconclusive trials in the bottom two rows, respectively.

**Table 1:** Probability of concluding each hypothesis, average sample size and percentage of inconclusive trials (toxicity probability = 0.15)

**Scenario**	**BHT-A**	**BHT-B**	**indep**	**BMA**	**BIT**	**Scenario**	**BHT-A**	**BHT-B**	**indep**	**BMA**	**BIT**
**0.1 *& * 0.1**						**0.1 *& * 0.3**					
P(*H*_0_)	0.936	0.935	0.894	0.94	0.935	P(*H*_0_)	0.258	0.295	0.277	0.396	0.355
P(*H*_1_)	0.041	0.049	0.057	0.037	0.039	P(*H*_1_)	0.604	0.645	0.676	0.435	0.501
P(*H*_2_)	0.022	0.014	0.003	0.018	0.016	P(*H*_2_)	0.085	0.029	0.02	0.03	0.051
P(*H*_3_)	0	0	0	0.003	0.01	P(*H*_3_)	0.045	0.024	0.012	0.129	0.088
P($H_{2}^{⋆}$)	0.022	0.014	0.003	0.021	0.026	P($H_{2}^{⋆}$)	0.13	0.053	0.033	0.159	0.139
avg ss	25.209	25.205	24.985	24.709	24.621	avg ss	26.862	26.96	25.328	26.711	25.606
*%* inconclusive	0.001	0.002	0.046	0.002	0	*%* inconclusive	0.008	0.007	0.014	0.01	0.005
**0.2 *& * 0.2**						**0.1 *& * 0.4**					
P(*H*_0_)	0.434	0.524	0.401	0.519	0.496	P(*H*_0_)	0.092	0.092	0.077	0.171	0.136
P(*H*_1_)	0.133	0.183	0.225	0.106	0.115	P(*H*_1_)	0.765	0.84	0.877	0.553	0.723
P(*H*_2_)	0.386	0.246	0.104	0.267	0.255	P(*H*_2_)	0.06	0.029	0.016	0.017	0.018
P(*H*_3_)	0.036	0.029	0.025	0.088	0.129	P(*H*_3_)	0.081	0.037	0.027	0.247	0.123
P($H_{2}^{⋆}$)	0.422	0.275	0.129	0.355	0.384	P($H_{2}^{⋆}$)	0.141	0.066	0.042	0.264	0.141
avg ss	26.782	27.805	26.341	26.919	25.995	avg ss	25.871	25.68	24.84	27.431	25.754
*%* inconclusive	0.011	0.018	0.245	0.02	0.005	*%* inconclusive	0.002	0.002	0.004	0.012	0
**0.1 *& * 0.2**						**0.3 *& * 0.4**					
P(*H*_0_)	0.577	0.619	0.609	0.701	0.674	P(*H*_0_)	0.03	0.031	0.024	0.04	0.04
P(*H*_1_)	0.326	0.336	0.341	0.203	0.241	P(*H*_1_)	0.152	0.249	0.274	0.043	0.109
P(*H*_2_)	0.072	0.034	0.013	0.047	0.036	P(*H*_2_)	0.425	0.383	0.319	0.379	0.283
P(*H*_3_)	0.01	0.002	0.004	0.045	0.048	P(*H*_3_)	0.392	0.336	0.324	0.535	0.565
P($H_{2}^{⋆}$)	0.082	0.036	0.016	0.092	0.084	P($H_{2}^{⋆}$)	0.817	0.719	0.643	0.914	0.848
avg ss	27.881	27.231	25.673	25.704	25.426	avg ss	25.244	25.52	25.291	25.116	25.191
*%* inconclusive	0.015	0.009	0.034	0.004	0.001	*%* inconclusive	0.001	0.001	0.059	0.003	0.003
**0.3 *& * 0.3**						**0.3 *& * 0.5**					
P(*H*_0_)	0.085	0.108	0.086	0.124	0.108	P(*H*_0_)	0.006	0.004	0.005	0.006	0.011
P(*H*_1_)	0.104	0.192	0.211	0.061	0.081	P(*H*_1_)	0.179	0.295	0.293	0.041	0.133
P(*H*_2_)	0.618	0.557	0.355	0.503	0.455	P(*H*_2_)	0.233	0.2	0.193	0.236	0.162
P(*H*_3_)	0.187	0.141	0.141	0.3	0.353	P(*H*_3_)	0.581	0.501	0.496	0.714	0.689
P($H_{2}^{⋆}$)	0.805	0.698	0.496	0.803	0.808	P($H_{2}^{⋆}$)	0.814	0.701	0.689	0.95	0.851
avg ss	25.594	26.214	25.779	26.084	25.505	avg ss	24.74	25.202	25.063	24.832	25.374
*%* inconclusive	0.006	0.002	0.206	0.012	.003	*%* inconclusive	0.001	0	0.013	0.003	0.005
**0.4 *& * 0.4**						**0.4 *& * 0.5**					
P(*H*_0_)	0.006	0.019	0.009	0.012	0.013	P(*H*_0_)	0	0.001	0.002	0.002	0.002
P(*H*_1_)	0.032	0.078	0.103	0.011	0.03	P(*H*_1_)	0.049	0.087	0.11	0.011	0.029
P(*H*_2_)	0.611	0.589	0.497	0.573	0.487	P(*H*_2_)	0.336	0.335	0.34	0.405	0.319
P(*H*_3_)	0.351	0.314	0.319	0.404	0.47	P(*H*_3_)	0.615	0.577	0.533	0.582	0.65
P($H_{2}^{⋆}$)	0.962	0.903	0.816	0.977	0.957	P($H_{2}^{⋆}$)	0.951	0.912	0.874	0.987	0.969
avg ss	24.326	24.755	24.742	24.436	24.458	avg ss	24.289	24.408	24.514	24.319	24.359
*%* inconclusive	0	0	0.072	0	0	*%* inconclusive	0	0	0.014	0	0
**0.5 *& * 0.5**						**0.4 *& * 0.6**					
P(*H*_0_)	0	0	0	0	0	P(*H*_0_)	0.001	0.001	0	0.001	0
P(*H*_1_)	0.007	0.017	0.014	0.002	0.002	P(*H*_1_)	0.053	0.114	0.112	0.01	0.036
P(*H*_2_)	0.495	0.498	0.513	0.558	0.491	P(*H*_2_)	0.159	0.14	0.175	0.237	0.176
P(*H*_3_)	0.498	0.485	0.457	0.44	0.507	P(*H*_3_)	0.787	0.745	0.711	0.752	0.788
P($H_{2}^{⋆}$)	0.993	0.983	0.97	0.998	0.998	P($H_{2}^{⋆}$)	0.946	0.885	0.886	0.989	0.964
avg ss	24.028	24.188	24.21	24.025	24.072	avg ss	24.193	24.491	24.412	24.177	24.341
*%* inconclusive	0	0	0.016	0	0	*%* inconclusive	0	0	0.002	0	0

In all scenarios, there are few early terminations due to toxicity because the true probabilities of toxicities are assumed low and the prior distributions for toxicity are informative (summary not shown). In the first scenario, *H*_0_ is true. The five designs are tuned to result in similar probabilities of declaring *H*_0_ (0.936, 0.935, 0.894, 0.94, and 0.935, respectively) and the corresponding average sample sizes, with the independent BHT design performing a little worse. In the second scenario, although the true response rate of 0.2 is between the null value 0.1 and the target value 0.3, it may be desirable to claim *H*_0_ because neither dose achieves the target response rate (but this may be debatable). The BHT-B and BMA designs result in higher probabilities of claiming *H*_0_, and use slightly larger sample sizes. The independent BHT design performs the worst. In the third scenario, i.e., 0.1 & 0.2, we may similarly want to claim *H*_0_. BMA and BIT yield the highest probabilities of claiming *H*_0_, and also use fewer patients than the joint BHT. In the next three scenarios, *H*_2_ is true. The probability of concluding $H_{2}^{⋆}$ is higher under BHT-A, BMA, and BIT. BHT-A has the largest probability of correctly claiming *H*_2_ under scenarios 0.3 & 0.3 and 0.4 & 0.4, and BMA has the largest probability of claiming *H*_2_ under scenario 0.5 & 0.5. The results in these three scenarios highlight the advantages of using nonlocal alternative priors in BHT and BMA, as these priors are perceived to be helpful in identifying equality of response rates between doses. In the next two scenarios, *H*_1_ is true. The independent BHT and BHT-B designs lead to the highest probabilities of claiming *H*_1_. The independent BHT design also uses the smallest sample sizes on average. In these two scenarios, BMA performs the worst, because the response rate estimates corresponding to the model in which the two response rates are equal are averaged in the final results, which decreased the higher response rate. In the last four scenarios, *H*_3_ is true. BMA yields the highest probability $p(H_{2}^{⋆})$ in all four scenarios. This may be explained by the fact that the incorrect model that assumes equality for the response rates (i.e., *M*_1_) in fact has strengthened the claim that both doses are promising. For 0.3 & 0.4 and 0.3 & 0.5, BMA and BIT yield the highest probabilities of claiming *H*_3_. And for 0.4 & 0.5 and 0.4 & 0.6, BHT-A and BIT result in the highest probabilities of *H*_3_. As expected, BHT-B leads to a smaller probability of $H_{2}^{⋆}$ than BHT-A. In all 12 scenarios, the inconclusive percentages are the highest under the independent BHT design.

The overall results suggest that the BHT-A design performs the best among all designs. The BMA design performs reasonably well (except in scenarios 0.1 & 0.3, and 0.1 & 0.4), similarly to or marginally better than the BIT design and independent BHT design. The BHT-B design tends to perform adequately in most scenarios, but not the best in any scenario. To summarize the robustness of the performances of all five designs, we counted the number of scenarios out of 10 scenarios (excluding scenarios 0.2 & 0.2, and 0.1 & 0.2) in which each design performs the best or almost the best (in terms of the percentage of drawing the correct conclusion) across all five designs and the number of scenarios in which the design performs inadequately (defined as when the chance of drawing a correct conclusion is at least 15 percentage points less than that of the best design for that scenario). For example, the corresponding numbers are (3,1) for the BHT-A design, meaning that in 3 out of the 10 scenarios, the design performs the best or nearly the best, and in 1 out of the 10 scenarios it performs inadequately. The corresponding pairs of numbers for the BHT-B, indep, BMA, and BIT designs are (0,2), (2,3), (2,2), and (3,2), respectively. We excluded scenarios 0.1 & 0.2 and 0.2 & 0.2 because it is unclear which conclusion should be deemed ‘correct’ in these scenarios. We re-counted these numbers by further excluding scenarios 0.3 & 0.4 and 0.4 & 0.5, because they represent minimal levels of clinically meaningful difference in the response rate between doses, and thus may be of less relevance than other scenarios. With these exclusions, the numbers are (3,0), (0,1), (2,2), (2,2), and (1,2) for the BHT-A, BHT-B, indep, BMA, and BIT designs, respectively. These results demonstrate the robust performance of the proposed BHT-A design.

### Comparison to the independent Simon optimal two-stage designs

We also compared our proposed Bayesian designs to the independent Simon optimal two-stage designs, perhaps the most commonly used design for single-arm phase II trials. Since we have demonstrated that the BHT-A design performs more robustly than the BMA and BHT-B designs, we compare only the BHT-A and independent Simon two-stage designs. We conduct this comparison separately because the Simon design does not include early stopping for efficacy. For simplicity, we assumed toxicity was low at both dose levels again, and only considered efficacy.

We extended the Simon optimal two-stage design to a setting with two doses, as follows. First, we applied the two-stage design to each dose level independently. We aimed to control the type I error to be 0.05 when both response rates were 0.1 and the type II error to be 0.2 when both response rates were 0.3. So at each dose, the type I error was chosen to be 0.0253 and the type II error was 0.106. Under the optimality criterion of Simon, the required maximum sample size for each dose was 45, with 17 in the first stage. We concluded *H*_0_ if both doses were rejected, *H*_1_ if only the lower dose was rejected, and $H_{2}^{*}$ if neither dose was rejected. Here rejection of a dose means that the dose is not considered to be promising (or hypothesis *θ*_1 _= *θ*_0_ or *θ*_2 _= *θ*_0_ is concluded). If $H_{2}^{⋆}$ was concluded, we claimed *H*_2_ if *RR*_1 _≥ *RR*_2 _- *δ*, and *H*_3_ otherwise, where *RR*_1_ and *RR*_2_ were the observed proportions of patients who experienced efficacy at lower and higher doses, respectively. We first used *δ *= 0.05, and performed additional sensitivity analyses using *δ *= 0.03 and 0.07. If only the higher dose was rejected, the trial was claimed to be inconclusive. The reasons why we compare with the independent Simon’s designs are: 1) Based on our experiences at MD Anderson Cancer Center, it is a commonly used approach in designing phase II oncology trials with more than one dose groups, even under a plausible assumption that the response rates are ordered between dose levels; 2) we are not aware of a published version of the Simon optimal two-stage designs for ordered dose groups in the literature.

To make the BHT-A design comparable with the Simon two-stage designs, we modified our monitoring rule to allow for early stopping only for futility. Specifically, we terminated the trial if *p *(*H*_0_|*x*) was above 0.848, and closed the lower dose arm if *p *(*H*_1_|*x*) was above 0.848. The BHT-A design utilized continuous monitoring after the outcomes of a minimum of 24 patients across both doses had been observed. The maximum sample size was also set to be 45 at each dose level. At the end of the trial, we claimed *H*_0_, *H*_1_, or $H_{2}^{⋆}$ if the corresponding posterior probability was above 0.5. If $H_{2}^{⋆}$ was claimed, we concluded *H*_2_ if *p *(*x*|*H*_2_)/*p *(*x*|*H*_3_) > 1.37, and concluded *H*_3_ otherwise.

We considered the same 12 scenarios, and under each scenario we simulated 1,000 trials. The operating characteristics of both designs are shown in Tables 2, with *δ *= 0.05, 0.03, and 0.07 for the Simon designs labeled as ‘Simon I’, ‘Simon II’, and ‘Simon III’, respectively. The column ‘SS’ shows the average total sample size; columns ‘SS 1’ and ‘SS 2’ show the average sample size for the lower and higher doses, respectively. The type I error rate was slightly lower under BHT. For scenario 0.3 & 0.3, the BHT design performed much better than the Simon I design, with 14*%* higher $p(H_{2}^{⋆})$ and 35*%* higher *P*(*H*_2_). For scenario 0.1 & 0.3, BHT resulted in a little lower *P*(*H*_1_) than Simon I. In other scenarios, BHT-A and Simon I designs perform comparably. In scenario 0.1 & 0.1 where neither dose level is promising, BHT-A required at least six patients fewer at each dose compared to the Simon I design. The comparisons with the Simon II and Simon III designs are similar. In summary, compared with the Simon optimal two-stage designs, our proposed BHT-A design can terminate trials of unpromising doses early, by utilizing a continuous monitoring rule and nonlocal alternative prior distributions in the hypothesis tests.

**Table 2:** Comparisons of BHT-A and Simon optimal two-stage designs

	***P*** **(*H***_**0**_**)**	***P*** **(*H***_**1**_**)**	***P*** **(*H***_**2**_**)**	***P*** **(*H***_**3**_**)**	$P\left(H_{2}^{⋆}\right)$	**SS**	**SS 1**	**SS 2**	***%* incon**
					**0.1 & 0.1**				
BHT-A	0.978	0.014	0.003	0.000	0.003	34.5	17.1	17.4	0.005
Simon I	0.954	0.022	0.001	0.000	0.001	47.1	23.6	23.5	0.022
Simon II	0.954	0.022	0.001	0.000	0.001	47.1	23.6	23.5	0.022
Simon III	0.954	0.022	0.001	0.000	0.001	47.1	23.6	23.5	0.022
					**0.2 & 0.2**				
BHT-A	0.408	0.142	0.408	0.003	0.411	68.6	33.3	35.3	0.039
Simon I	0.261	0.250	0.203	0.040	0.243	73.2	36.7	36.5	0.246
Simon II	0.261	0.250	0.174	0.069	0.243	73.2	36.7	36.5	0.246
Simon III	0.261	0.250	0.221	0.022	0.243	73.2	36.7	36.5	0.246
					**0.1 & 0.2**				
BHT-A	0.623	0.343	0.027	0.000	0.027	53.6	23.6	30.0	0.007
Simon I	0.515	0.457	0.008	0.004	0.012	60.0	23.7	36.3	0.016
Simon II	0.515	0.457	0.008	0.004	0.012	60.0	23.7	36.3	0.016
Simon III	0.515	0.457	0.011	0.001	0.012	60.0	23.7	36.3	0.016
					**0.3 & 0.3**				
BHT-A	0.039	0.056	0.773	0.132	0.905	86.7	42.8	43.9	0.000
Simon I	0.008	0.094	0.574	0.220	0.794	85.5	42.9	42.6	0.104
Simon II	0.008	0.094	0.506	0.288	0.794	85.5	42.9	42.6	0.104
Simon III	0.008	0.094	0.632	0.162	0.794	85.5	42.9	42.6	0.104
					**0.4 & 0.4**				
BHT-A	0.002	0.008	0.706	0.284	0.990	89.7	44.7	44.9	0.000
Simon I	0.000	0.014	0.675	0.300	0.976	89.3	44.6	44.7	0.010
Simon II	0.000	0.014	0.588	0.387	0.976	89.3	44.6	44.7	0.010
Simon III	0.000	0.014	0.748	0.227	0.976	89.3	44.6	44.7	0.010
					**0.5 & 0.5**				
BHT-A	0.000	0.001	0.659	0.340	0.999	90.0	45.0	45.0	0.000
Simon I	0.000	0.001	0.691	0.305	0.996	89.9	45.0	44.9	0.003
Simon II	0.000	0.001	0.626	0.370	0.996	89.9	45.0	44.9	0.003
Simon III	0.000	0.001	0.762	0.234	0.996	89.9	45.0	44.9	0.003
					**0.1 & 0.3**				
BHT-A	0.150	0.811	0.030	0.006	0.036	64.9	23.9	41.0	0.003
Simon I	0.104	0.872	0.006	0.016	0.022	66.4	23.6	42.8	0.002
Simon II	0.104	0.872	0.002	0.020	0.022	66.4	23.6	42.8	0.002
Simon III	0.104	0.872	0.007	0.015	0.022	66.4	23.6	42.8	0.002
					**0.1 & 0.4**				
BHT-A	0.021	0.949	0.007	0.023	0.030	65.2	20.9	44.4	0.000
Simon I	0.013	0.957	0.001	0.028	0.029	68.5	23.9	44.6	0.001
Simon II	0.013	0.957	0.000	0.028	0.029	68.5	23.9	44.6	0.001
Simon III	0.013	0.957	0.002	0.027	0.029	68.5	23.9	44.6	0.001
					**0.3 & 0.4**				
BHT-A	0.006	0.097	0.358	0.539	0.897	87.5	42.7	44.8	0.000
Simon I	0.000	0.100	0.336	0.556	0.892	87.7	42.9	44.8	0.008
Simon II	0.000	0.100	0.261	0.631	0.892	87.7	42.9	44.8	0.008
Simon III	0.000	0.100	0.412	0.480	0.892	87.7	42.9	44.8	0.008
					**0.3 & 0.5**				
BHT-A	0.000	0.116	0.071	0.813	0.884	87.3	42.3	45.0	0.000
Simon I	0.000	0.104	0.075	0.820	0.896	87.9	43.0	45.0	0.001
Simon II	0.000	0.104	0.050	0.846	0.896	87.9	43.0	45.0	0.001
Simon III	0.000	0.104	0.122	0.774	0.896	87.9	43.0	45.0	0.001
					**0.4 & 0.5**				
BHT-A	0.000	0.010	0.289	0.701	0.990	89.7	44.7	45.0	0.000
Simon I	0.000	0.014	0.353	0.632	0.984	89.6	44.6	45.0	0.000
Simon II	0.000	0.014	0.280	0.705	0.984	89.6	44.6	45.0	0.000
Simon III	0.000	0.014	0.424	0.560	0.984	89.6	44.6	45.0	0.000
					**0.4 & 0.6**				
BHT-A	0.000	0.021	0.051	0.928	0.979	89.4	44.4	45.0	0.000
Simon I	0.000	0.016	0.074	0.910	0.984	89.6	44.6	45.0	0.000
Simon II	0.000	0.016	0.051	0.934	0.984	89.6	44.6	45.0	0.000
Simon III	0.000	0.016	0.116	0.868	0.984	89.6	44.6	45.0	0.000

Note: A tuning parameter *δ*= 0.05, 0.03, or 0.07 was used in the Simon I, II, or III design, respectively.

To compare the robustness of the performances of all four designs, i.e., BHT-A and Simon I, II and III designs, we similarly report the numbers of scenarios in which each design performs the best and inadequately. The pairs of numbers are (5,0), (2,1), (6,2), and (4,1) out of 10 scenarios, and (4,0), (2,1), (4,2), and (4,0) out of the 8 scenarios, for the BHT-A, Simon I, II, and III designs, respectively, with the same 10 and 8 scenarios selected in Section “Design operating characteristics”. We extended the definitions for ‘best’ and ‘inadequate’ by also accounting for situations where an average total sample size is reduced by 10 or more when the percentages of drawing the correct conclusion are similar (i.e., scenario 0.1 & 0.1). These results suggest that the proposed BHT-A design performs more robustly than the three versions of the independent Simon optimal two-stage designs.

## Conclusions

We have proposed two Bayesian designs for phase II oncology trials to assess the efficacy of more than one dose level of a treatment and identify the MaxED, one using Bayesian hypothesis tests and the other using Bayesian model averaging. Both designs use the recently developed nonlocal alternative priors for the response rates. Our simulation results suggest that the BHT-A design performs better overall than the BMA design, the independent single-arm design using Bayesian hypothesis tests with a nonlocal alternative prior, and the Bayesian isotonic regression transformation (BIT)-based design. The better performance of the BHT-A design compared to the BMA design is consistent with our expectation that the Bayesian hypothesis test approach outperforms the posterior credible interval-based approach, the latter being used in the BMA design. Of the two versions of the BHT design, BHT-A (assuming equal prior probability of hypotheses *H*_0_ through *H*_3_) outperforms BHT-B (assuming equal prior probability of hypotheses *H*_0_, *H*_1_ and $H_{2}^{⋆}$), if we consider the scenarios in which both doses are effective to be more likely in practice. Our additional simulation results similarly show that the BHT-A design that is modified to only allow for futility monitoring performs better than the independent Simon optimal two-stage designs. These results demonstrate the expected advantages of using Bayesian hypothesis tests with nonlocal alternative priors to continuously monitor the trial and identify the MaxED. Specifically, as one of our goals is to determine whether a lower dose may result in the same efficacy as a higher dose does, the nonlocal alternative priors allow the trial to accumulate strong evidence in favor of the null hypothesis of equal response rates between doses when this hypothesis holds.

The proposed designs allow the user to specify the values of *τ*_1_ and *τ*_2_ to reflect the physician’s notion of what is meant by a clinically meaningful difference in the response rate both from historical control and between dose groups. The maximum sample size *N* is often chosen based on both practical considerations such as the budget constraints and the physician’s judgment on the trade-off between the gain in the probability of drawing the correct conclusion and the time and resources required to achieve such a gain. Simulations are used to evaluate the trade-off, as well as to select other design parameters. When more than three doses are evaluated, our proposed designs are still applicable, but the computational burden increases.

Our proposed designs use continuous monitoring. To be more practical, our designs can be modified to allow for semi-continuous monitoring, e.g., monitoring after the outcomes of every five patients are observed. Our designs can be extended to trials that assume an umbrella ordering of efficacy across doses [25], with the same goal of identifying the MaxED. In that case, different hypotheses that correspond to dose-response curves with different peak locations can be defined and similarly modeled using nonlocal alternative priors.

The designs we propose assume equal randomization across doses. However, this is not required. Adaptive randomization may be implemented, with the randomization probabilities depending on the current overall comparison of efficacy across doses. However, as mentioned in Section “Bayesian hypothesis testing”, the gain by using outcome-adaptive randomization may not be substantial for trials with two or three arms.

Both proposed designs require a prior specification of the model probabilities. In our simulations, we assumed equal prior model probabilities to represent prior ignorance. If there is *a priori* a higher probability of a certain model being true, the performances of the proposed designs may be improved by assigning unbalanced prior model probabilities.

## Abbreviations

MaxED: Maximum effective dose; MTA: Molecularly targeted agent; MTD: Maximum tolerated dose; LOXL2: Lysyl oxidase homolog 2; DLT: Dose-limiting toxicity; BHT: Bayesian hypothesis testing; BMA: Bayesian model averaging; BIT: Bayesian isotonic regression transformation.

## Competing interests

Both authors declare that they have no competing interests.

## Authors’ contributions

BG implemented the simulation study, interpreted the results, and drafted the manuscript. YL conceived of the study and edited the manuscript. Both authors read and approved the final manuscript.

## Pre-publication history

The pre-publication history for this paper can be accessed here:

http://www.biomedcentral.com/1471-2288/14/95/prepub
